# Supplementary material for: Coregistration of heading to visual cues in retrosplenial cortex
Source: Nat Commun. 2023 Apr 8;14:1992. doi: 10.1038/s41467-023-37704-5 (PMC10082791; doi:10.1038/s41467-023-37704-5)
Supplement: Supplementary file 1 — Supplementary Information [file 41467_2023_37704_MOESM1_ESM.pdf]

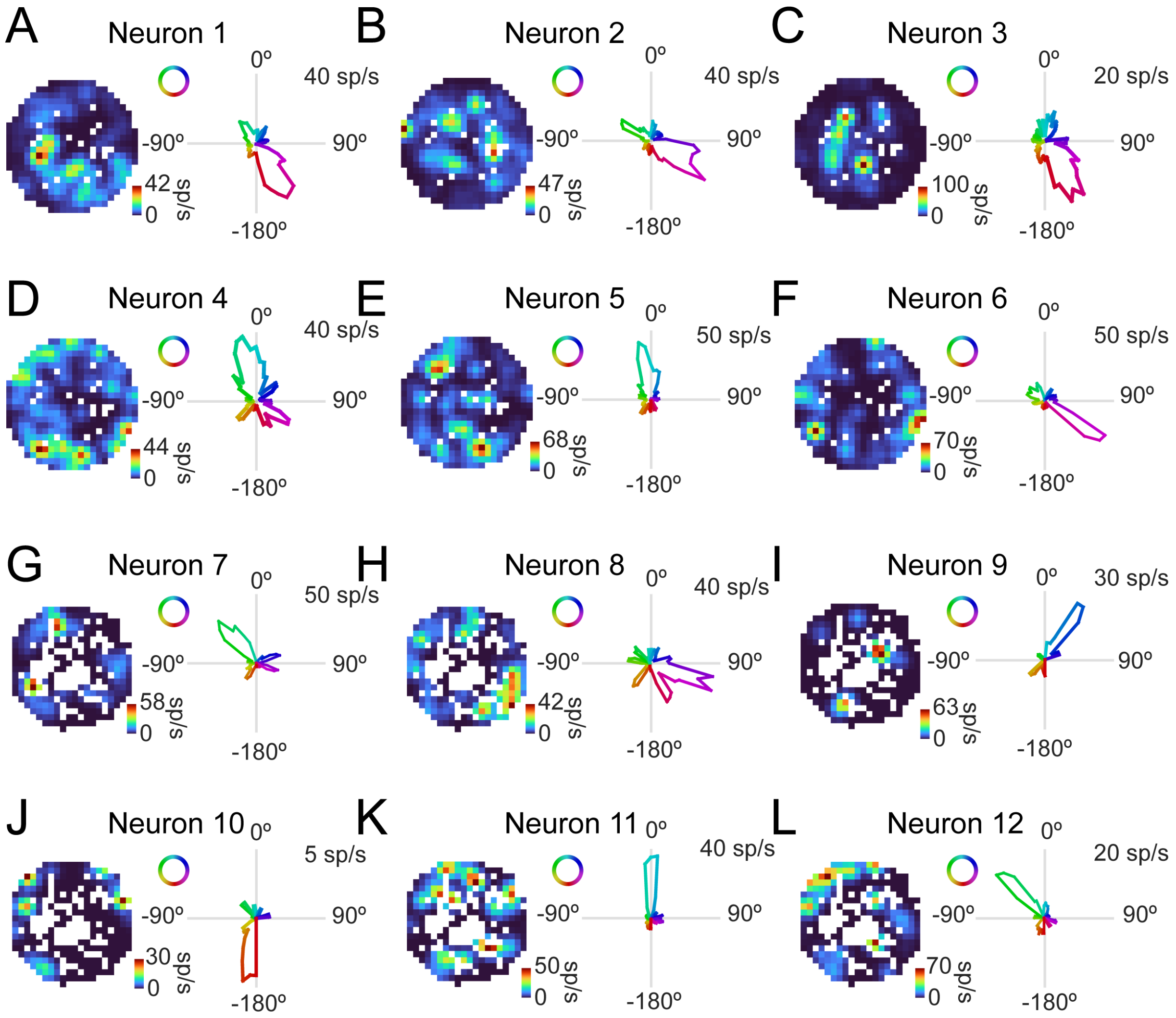

Figure S1: RSC neurons represent heading during locomotion in the floating chamber.
A) *Left:* Example spike rate heat map of a single neuron, showing the neuron's response as chamber moves around the mouse. *Right:* Example tuning curve from the same neuron.
B-L) Same as **A** for additional example neurons.

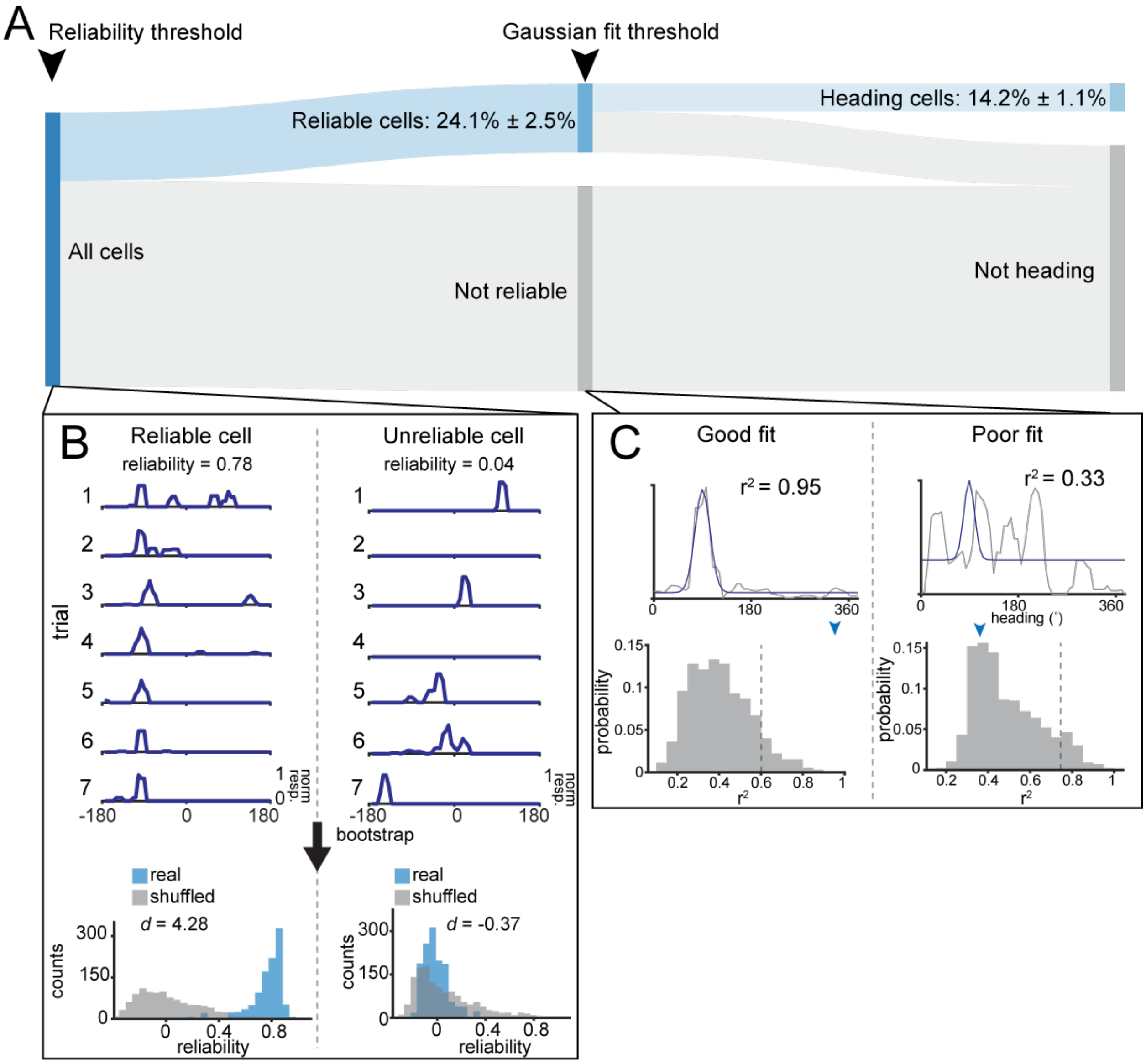

Figure S2: Schematic of procedure for determining whether a cell is heading responsive.

A) Sankey diagram showing the percentage of cells passing each selection criteria for determining heading responsiveness. All cells first pass through a reliability threshold, followed by a Gaussian fit threshold. Both thresholds were calculated via a shuffling procedure (see Methods). Only cells which pass both thresholds were included in analyses.

B) Schematized reliability threshold using an example cell. *Left:* Trial-by-trial tuning curves for a reliable neuron showing a prominent peak in the same location over trials. Below are histograms comparing bootstrapped reliability of this cell versus a shuffled distribution. *Right:* Same as *left*, but for an unreliable cell.

C) Schematized procedure for Gaussian fit threshold. *Left:* Cells are fit with a single term Gaussian. A shuffled distribution of r^2 is created by circularly shifting tuning curves from each trial (see Methods). The distributions of r^2 are shown with a dotted line denoting the 99th percentilie and the blue arrow showing the reliability of the current cell. *Right:* Same as *left*, but for a poorly fit cell.

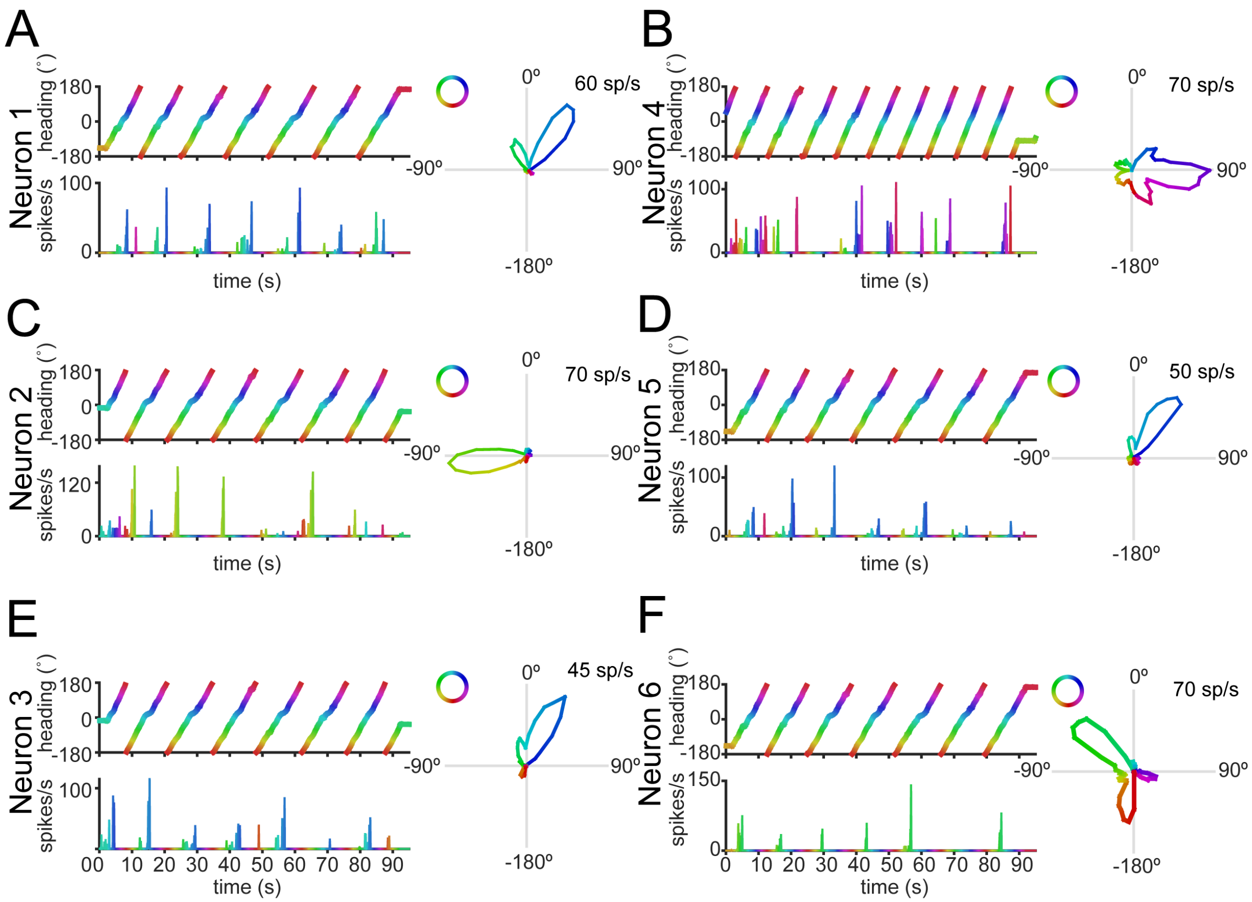

Figure S3: Neurons in the controlled rotation condition represent heading faithfully over multiple trials.
A) *Left, top:* Recorded heading over time, color coded by the current heading. *Left, bottom:* An example neuron's spike rate over the same duration, with the color of the line matched to the heading shown above. The similar color of the peaks show that the neuron is responding to the same heading on each trial. *Right:* Tuning curve from the example neuron constructed across all trials. For clarity, only a single block of consecutive trials is shown here for each neuron.
B-F) Same as **A** for additional example neurons.

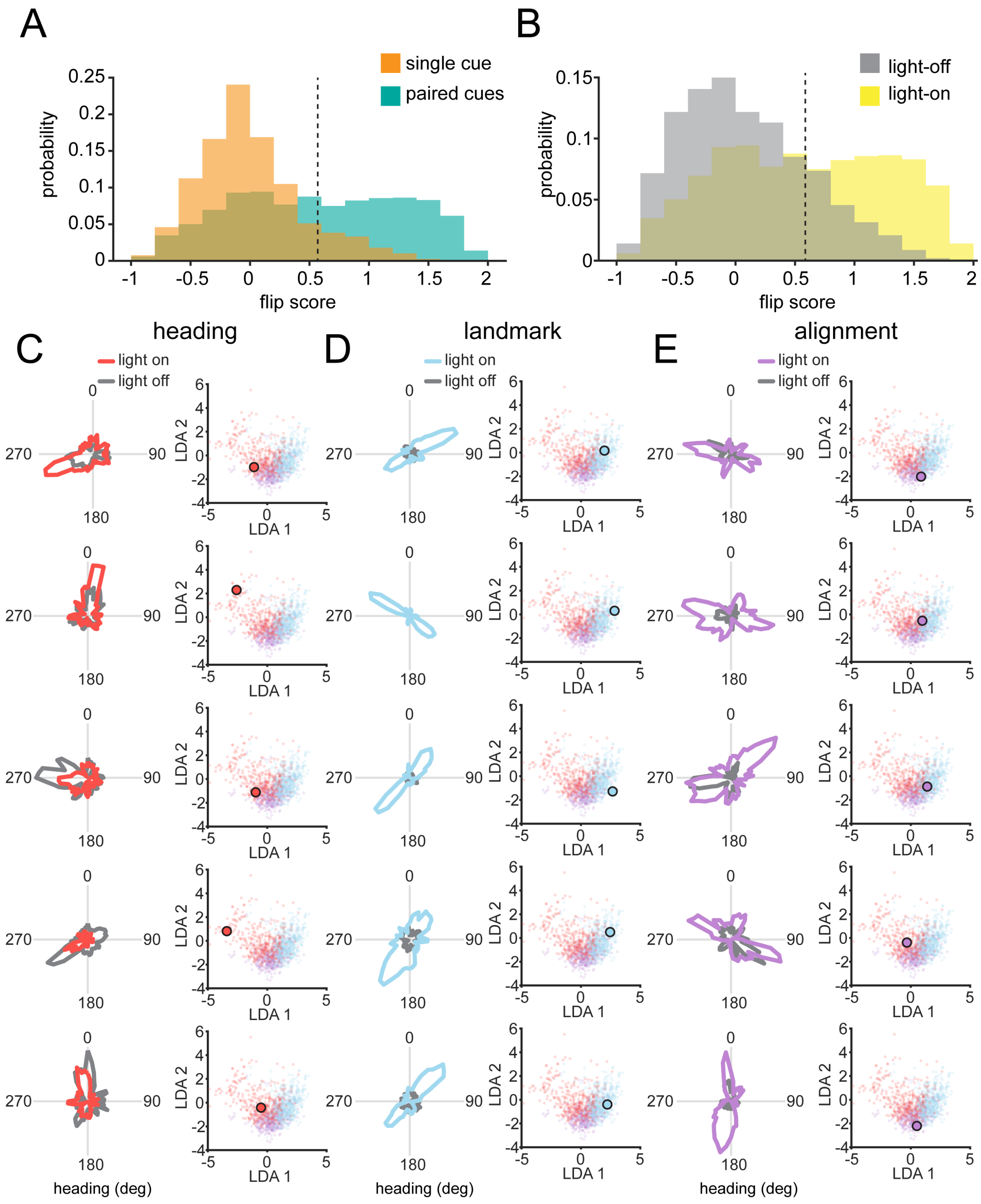

Figure S4: Neurons in the RSC have responses that cluster into three distinct profiles.
A) Histogram of flip scores between the single cue experiments (orange, Figure 1) and paired cue experiments (green).

B) Histogram of flip scores in the paired cue experiments comparing light-on (green) and light-off (gray) conditions.
C) Example tuning curves for heading cells, next to their position on the reduced dimensional space scatter plot. Each cell's position is shown with a large outlined dot.
D) Same as **A**, but for landmark cells.
E) Same as **A**, but for alignment cells.

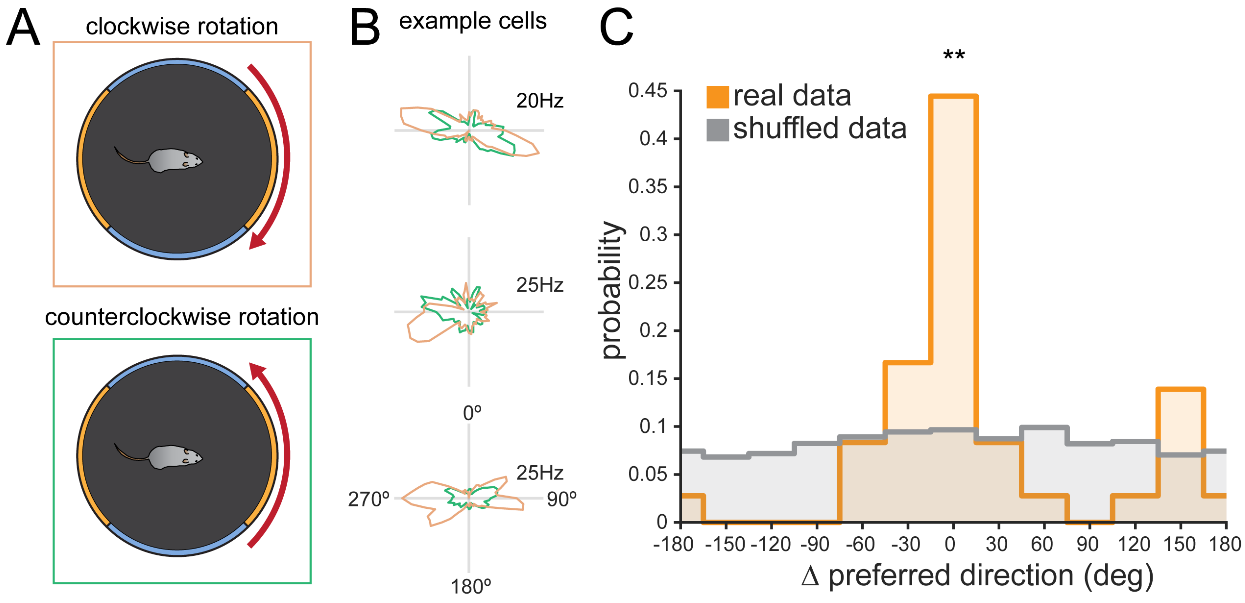

Figure S5: Heading neurons responses are conserved independent of rotation direction.
A) Schematic showing clockwise (orange) vs counterclockwise (green) rotations.
B) Three example neurons' responses to clockwise and counterclockwise rotations of the arena.
C) Histogram showing the differences in preferred directions for clockwise and counterclockwise rotations of the arena. $p = 0.0094$, bootstrapped KS-test. **: $p < 0.01$

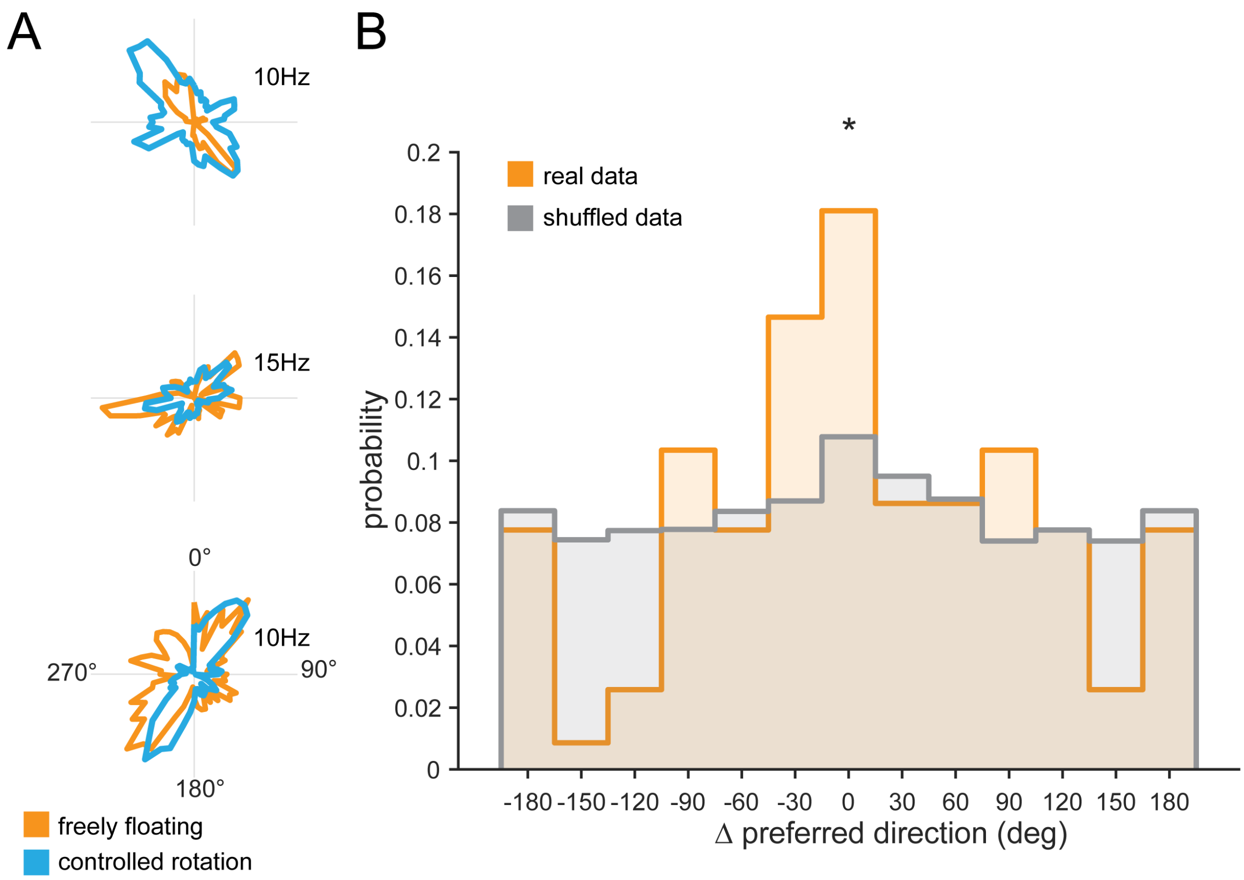

Figure S6: Heading neurons retain responses in controlled rotation versus free floating conditions on the floating platform.
A) Three example neurons' responses to freely floating (orange) and controlled rotations (blue) of the arena.
B) Histogram of differences of preferred direction between freely floating and controlled rotations, compared against a shuffled distribution. $p = 0.0310$ bootstrapped KS-test. *: $p < 0.05$

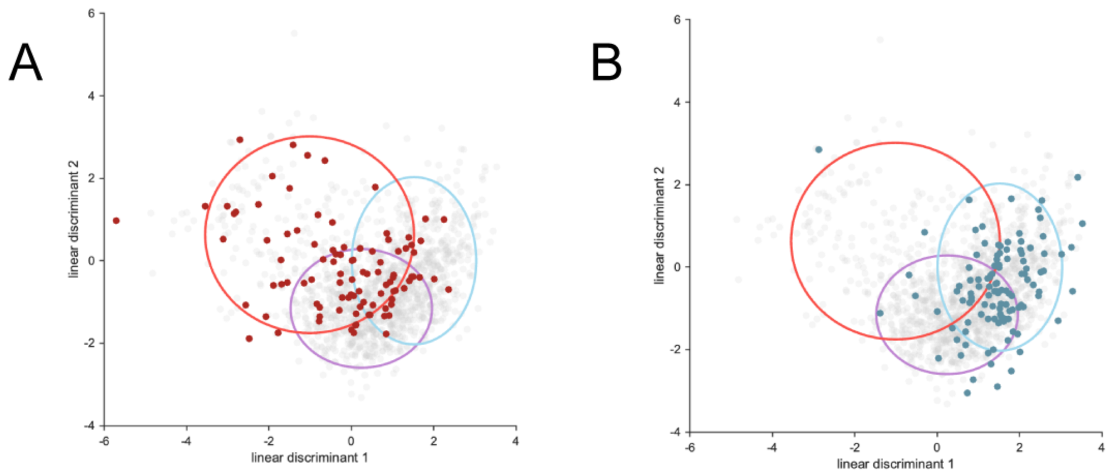

Figure S7: Axonal data in reduced dimensional space, separated by axon type.
A) ADN axons (red) plotted on top of the positions of RSC soma (gray) Colored circles represent the 95% CI ellipses surrounding each cluster (heading: red, visual: blue, purple: alignment).
B) Visual axons (blue) plotted on top of the positions of RSC soma (gray) Colored circles represent the 95% CI ellipses surrounding each cluster (heading: red, visual: blue, purple: alignment).

		Light on	Light off
	**All**	$< 0.0001^*$	0.4216
**Heading vs.**	**Landmark**	$< 0.0001^*$	0.0552
	**Alignment**	0.0374^*	0.2496
	**All**	0.7544	0.0214^*
**Landmark vs.**	**Heading**	$< 0.0001^*$	0.0552
	**Alignment**	0.0914	0.0058^*
	**All**	0.0214^*	0.5120
**Alignment vs.**	**Heading**	0.0374^*	0.2496
	**Landmark**	0.0914	0.0058^*

Table S1: Table of p-values for decoder comparisons across cell class.
Decoder performance for each cell class is compared to all other classes in a bootstrapped t-test (see Methods). Significant results are marked with an asterisk.
