# Supplementary material for: Coregistration of heading to visual cues in retrosplenial cortex
Source: Nat Commun. 2023 Apr 8;14:1992. doi: 10.1038/s41467-023-37704-5 (PMC10082791; doi:10.1038/s41467-023-37704-5)
Supplement: Supplementary file 3 — Reporting Summary [file 41467_2023_37704_MOESM3_ESM.pdf]

nature portfolio

Corresponding author(s): Michael Goard

Last updated by author(s): Mar 16, 2023

Reporting Summary

Nature Portfolio wishes to improve the reproducibility of the work that we publish. This form provides structure for consistency and transparency in reporting. For further information on Nature Portfolio policies, see our Editorial Policies and the Editorial Policy Checklist.

Statistics

For all statistical analyses, confirm that the following items are present in the figure legend, table legend, main text, or Methods section.

n/a	Confirmed	
☐	☒	The exact sample size (*n*) for each experimental group/condition, given as a discrete number and unit of measurement
☐	☒	A statement on whether measurements were taken from distinct samples or whether the same sample was measured repeatedly
☐	☒	The statistical test(s) used AND whether they are one- or two-sided<br>*Only common tests should be described solely by name; describe more complex techniques in the Methods section.*
☒	☐	A description of all covariates tested
☐	☒	A description of any assumptions or corrections, such as tests of normality and adjustment for multiple comparisons
☐	☒	A full description of the statistical parameters including central tendency (e.g. means) or other basic estimates (e.g. regression coefficient) AND variation (e.g. standard deviation) or associated estimates of uncertainty (e.g. confidence intervals)
☐	☒	For null hypothesis testing, the test statistic (e.g. *F*, *t*, *r*) with confidence intervals, effect sizes, degrees of freedom and *P* value noted<br>*Give P values as exact values whenever suitable.*
☒	☐	For Bayesian analysis, information on the choice of priors and Markov chain Monte Carlo settings
☐	☒	For hierarchical and complex designs, identification of the appropriate level for tests and full reporting of outcomes
☐	☒	Estimates of effect sizes (e.g. Cohen's *d*, Pearson's *r*), indicating how they were calculated

Our web collection on statistics for biologists contains articles on many of the points above.

Software and code

Policy information about availability of computer code

Data collection	MATLAB R2020a (Mathworks), Prairie View V5.4 (Bruker), Neurotar Tracking Software v2.2.1
Data analysis	Suite2p (https://github.com/MouseLand/suite2p), MATLAB R2020a (Mathworks), Github: https://github.com/ucsb-goard-lab/Neurotar-HD-Experiments, https://github.com/ucsb-goard-lab/HeadingDecoder, https://github.com/ucsb-goard-lab/Two-photon-calcium-post-processing

For manuscripts utilizing custom algorithms or software that are central to the research but not yet described in published literature, software must be made available to editors and reviewers. We strongly encourage code deposition in a community repository (e.g. GitHub). See the Nature Portfolio guidelines for submitting code & software for further information.

Data

Policy information about availability of data

All manuscripts must include a data availability statement. This statement should provide the following information, where applicable:

- Accession codes, unique identifiers, or web links for publicly available datasets
- A description of any restrictions on data availability
- For clinical datasets or third party data, please ensure that the statement adheres to our policy

All processed data and code will be shared on public repositories, links included in the Data Availability and Code Availability statements in the manuscript.

Human research participants

Policy information about studies involving human research participants and Sex and Gender in Research.

Reporting on sex and gender	NA
Population characteristics	NA
Recruitment	NA
Ethics oversight	NA

Note that full information on the approval of the study protocol must also be provided in the manuscript.

Field-specific reporting

Please select the one below that is the best fit for your research. If you are not sure, read the appropriate sections before making your selection.

☒ Life sciences ☐ Behavioural & social sciences ☐ Ecological, evolutionary & environmental sciences

For a reference copy of the document with all sections, see nature.com/documents/nr-reporting-summary-flat.pdf

Life sciences study design

All studies must disclose on these points even when the disclosure is negative.

Sample size	No a priori sample size calculation was performed. Sample sizes were chosen based on previous similar studies (Jacob et al 2017)
Data exclusions	For controlled rotation experiments, the experimental session was terminated if the mouse did not follow the rotation of the cage, and the subsequent recordings were discarded. For the head rotation vs. platform rotation experiments, recordings were discarded if they were unable to be derotated across the entire recording, a result of either poor recording quality or a misalignment between the center of the imaging plane and center of rotation. No other data were excluded.
Replication	All experiments were replicated in multiple mice (n = 26). Two-photon recordings were repeated within each mouse where indicated up to 4 nonoverlapping fields when the FOV was 829 x 829um or 5 nonoverlapping fields at 414 x 414um (for axonal recordings). All replications were successful after the exclusion criteria outlined above.
Randomization	Because we are not testing different experimental groups, no randomization was necessary in this study.
Blinding	Data collection and analyses were not performed blind because there were no experimental groups.

Reporting for specific materials, systems and methods

We require information from authors about some types of materials, experimental systems and methods used in many studies. Here, indicate whether each material, system or method listed is relevant to your study. If you are not sure if a list item applies to your research, read the appropriate section before selecting a response.

Materials & experimental systems

n/a	Involved in the study	
☒	☐	Antibodies
☒	☐	Eukaryotic cell lines
☒	☐	Palaeontology and archaeology
☐	☒	Animals and other organisms
☒	☐	Clinical data
☒	☐	Dual use research of concern

Methods

n/a	Involved in the study	
☒	☐	ChIP-seq
☒	☐	Flow cytometry
☒	☐	MRI-based neuroimaging

Animals and other research organisms

Policy information about studies involving animals; ARRIVE guidelines recommended for reporting animal research, and Sex and Gender in Research

Laboratory animals	26 mice (12 males, 14 females) aged 6 - 12 weeks. were used. All mice were Emx1-Cre (Jax Stock #005628) x ROSA-LNL-tTA (Jax Stock #011008) x TITL-GCaMP6s (Jax Stock #024104) or Slc17a7-Cre (Jax Stock #023527) x TITL2-GC6s-ICL-TTA2 (Jax Stock #031562). Mice were singly housed post-surgical procedure in a 12:12 light-dark cycle with the following controlled parameters: temperature (68-76F), humidity (30-70%), ventilation (10-15 ACH).
Wild animals	No wild animals were used.
Reporting on sex	No sex-based analyses were performed. All experiments were performed on mice of both sexes.
Field-collected samples	No field-collected samples were used.
Ethics oversight	All animal procedures were approved by the Institutional Animal Care and Use Committee at UC Santa Barbara.

Note that full information on the approval of the study protocol must also be provided in the manuscript.
